# The Affinity of Carboplatin to B-Vitamins and Nucleobases

**DOI:** 10.3390/ijms22073634

**Published:** 2021-03-31

**Authors:** Beata Szefler, Przemysław Czeleń, Przemysław Krawczyk

**Affiliations:** Department of Physical Chemistry, Faculty of Pharmacy, Collegium Medicum, Nicolaus Copernicus University, Kurpińskiego 5, 85-096 Bydgoszcz, Poland; przemekcz@cm.umk.pl (P.C.); przemekk@cm.umk.pl (P.K.)

**Keywords:** carboplatin, lung cancer, cancer treatment, vitamin B, thiamine, riboflavin, niacin, pyridoxal phosphate, adenine, guanine

## Abstract

Platinum compounds have found wide application in the treatment of various types of cancer and carboplatin is one of the main platinum-based drugs used as antitumor agents. The anticancer activity of carboplatin arises from interacting with DNA and inducing programmed cell death. However, such interactions may occur with other chemical compounds, such as vitamins containing aromatic rings with lone-pair orbitals, which reduces the anti-cancer effect of carboplatin. The most important aspect of the conducted research was related to the evaluation of carboplatin affinity to vitamins from the B group and the potential impact of such interactions on the reduction of therapeutic capabilities of carboplatin in anticancer therapy. Realized computations, including estimation of Gibbs Free Energies, allowed for the identification of the most reactive molecule, namely vitamin B6 (pyridoxal phosphate). In this case, the computational estimations indicating carboplatin reactivity were confirmed by spectrophotometric measurements.

## 1. Introduction

Carboplatin (1,1-cyclobutyldicarboxylate) is a drug used to treat cancer during chemotherapy [1]. It is a complex compound containing a heavy metal, i.e., platinum. The anticancer activity of cisplatin was discovered in the early 1960s by Professor Barnett Rosenberg at the University of Michigan [2,3].

Platinum compounds have found wide application in the treatment of various types of cancer and carboplatin is one of the main platinum-based drugs used as antitumor agents. It is intended specifically for the treatment of cancer of the testis, ovary, head, neck, and small cell lung cancer [4].

Currently, in addition to carboplatin, this group of drugs includes cisplatin and oxaliplatin. Carboplatin is a derivative of cisplatin and has a similar mechanism of action, differing only in terms of structure and toxicity [1]. Compared to cisplatin, carboplatin has a more favorable side effect profile. Carboplatin is also less reactive than cisplatin and, as a consequence, must be used at several times higher doses. Carboplatin is used as monotherapy or in combination with other anti-cancer drugs in the chemotherapy of small cell lung cancer [5,6] and advanced ovarian cancer [7,8].

**Figure 1:**
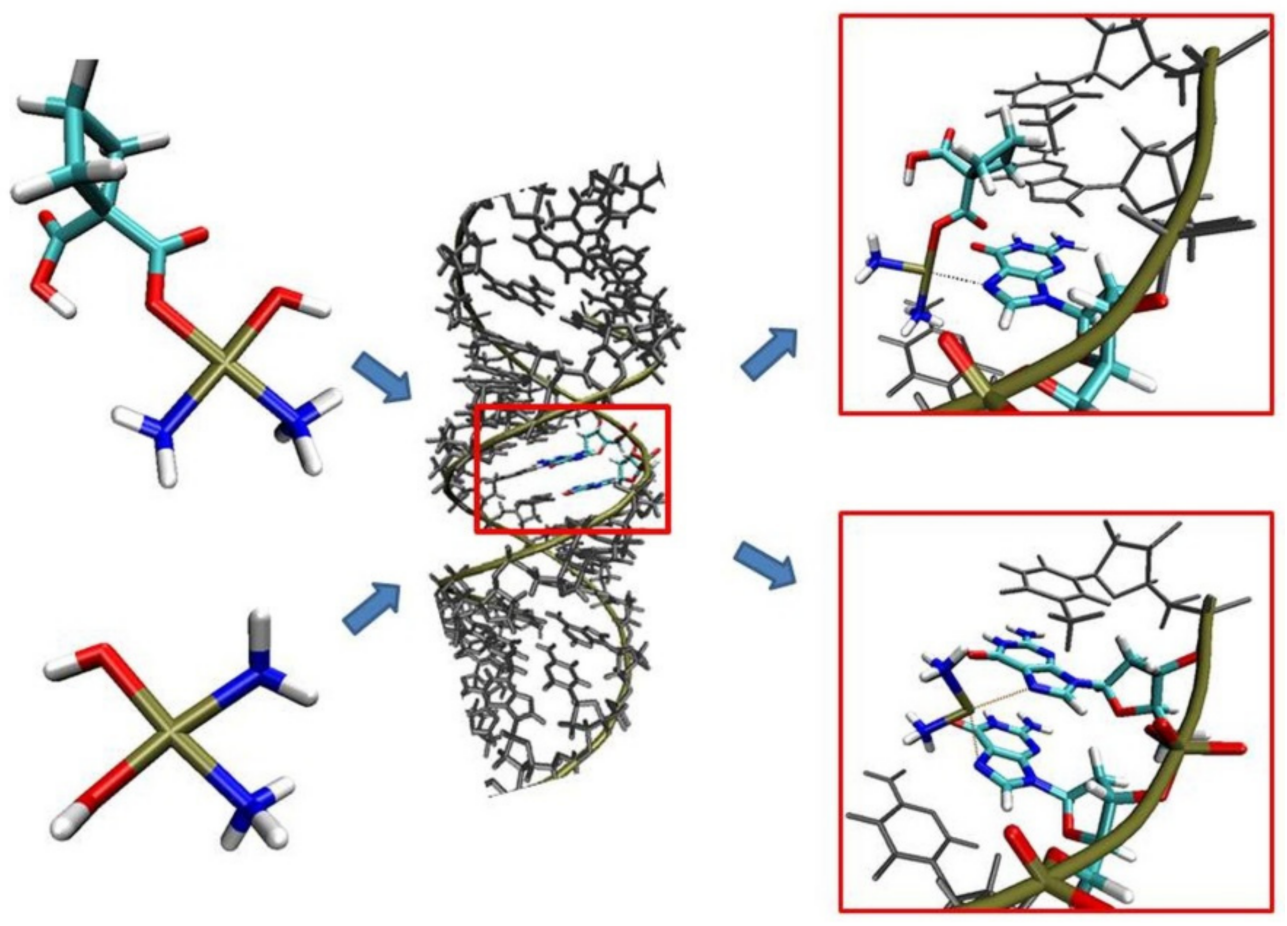
Formation of adducts between DNA and carboplatin.

Carboplatin, like other platinum compounds used to treat cancer, works by interacting with the cell’s genetic material, i.e., DNA nucleic acid (deoxyribonucleic acid). Platinum complexes react with DNA to form cross-links both within the molecule and between the DNA molecules. The adducts formed by this compound can be monoadducts or intra- and interchain diadducts [9] (Figure 1). The formation of abnormal bonds disturbs the structure of DNA, contributes to the formation of cracks in the DNA strand, interferes with the synthesis of DNA and RNA (ribonucleic acid), and prevents cell division [10]. The nature of these DNA adducts affects a number of transduction pathways and triggers apoptosis or necrosis in tumor cells. Inhibition of basic biological processes becomes a signal leading to cell death. The action of carboplatin relates particularly to rapidly dividing cells, which are cancer cells. Therefore, carboplatin limits the growth and development of cancer.

Carboplatin is less bound to plasma proteins than cisplatin. It bounds to several plasma proteins, including albumin, transferrin, and γ-globulin [11,12,13]. In contrast, nearly 90% of platinum-derived carboplatin binds to plasma proteins within 24 h after administration [13,14]. It is excreted mainly in the urine, and also in small amounts in bile.

### 1.1. Theoretical Studies

The anticancer activity of cisplatin was discovered for the first time in the 1960s by Professor Barnett Rosenberg at the University of Michigan [2,3]. Several years later the same scientist showed that the bivalent complex cis-[Pt(NH_3_)_2_Cl_2_] is much less active [2]. Carozzi showed that cisplatin has an anti-cancer effect [15]. After Rosenberg’s first work, other scientists found several thousands of platinum compounds, and some of them were synthesized, but in the end, only five of these compounds have been approved for commercialization: oxaliplatin, nedaplatin, lobaplatin, heptaplatin, and carboplatin [16]. Carboplatin (1,1-cyclobutyldicarboxylate), as one of the most often used antitumor compounds, is used first of all in the case of treatment of cancer of the testis, ovary, head, neck, and small cell lung cancer [4]. Brabec and Kasparkova described the effect of its activity on a molecular level [10]. Carboplatin binds to the nucleobases of DNA, which leads to suppression of replication and transcription, followed by cell death, apoptosis, or necrosis in tumor cells [10]. The adducts formed by this compound can be monoadducts or intra- and interchain diadducts [9] (Figure 1). Inducing greater damage to DNA, impairing mechanisms of DNA repair, or activating and preventing apoptosis may lead to decreased tumor cell viability [17,18]. Due to low toxicity, Kang and other scientists tested carboplatin on several tumors and the premise of these efforts was to determine against which tumors carboplatin can be effectively administered. It was found that these are: such as epithelial ovarian cancer, cancer of the esophagus, cancer of the cervix, lung cancer, cancer of the head and neck, breast cancer [19,20,21,22].

### 1.2. The Pathway of Carboplatin and Anticancer Activity

**Scheme 1:**
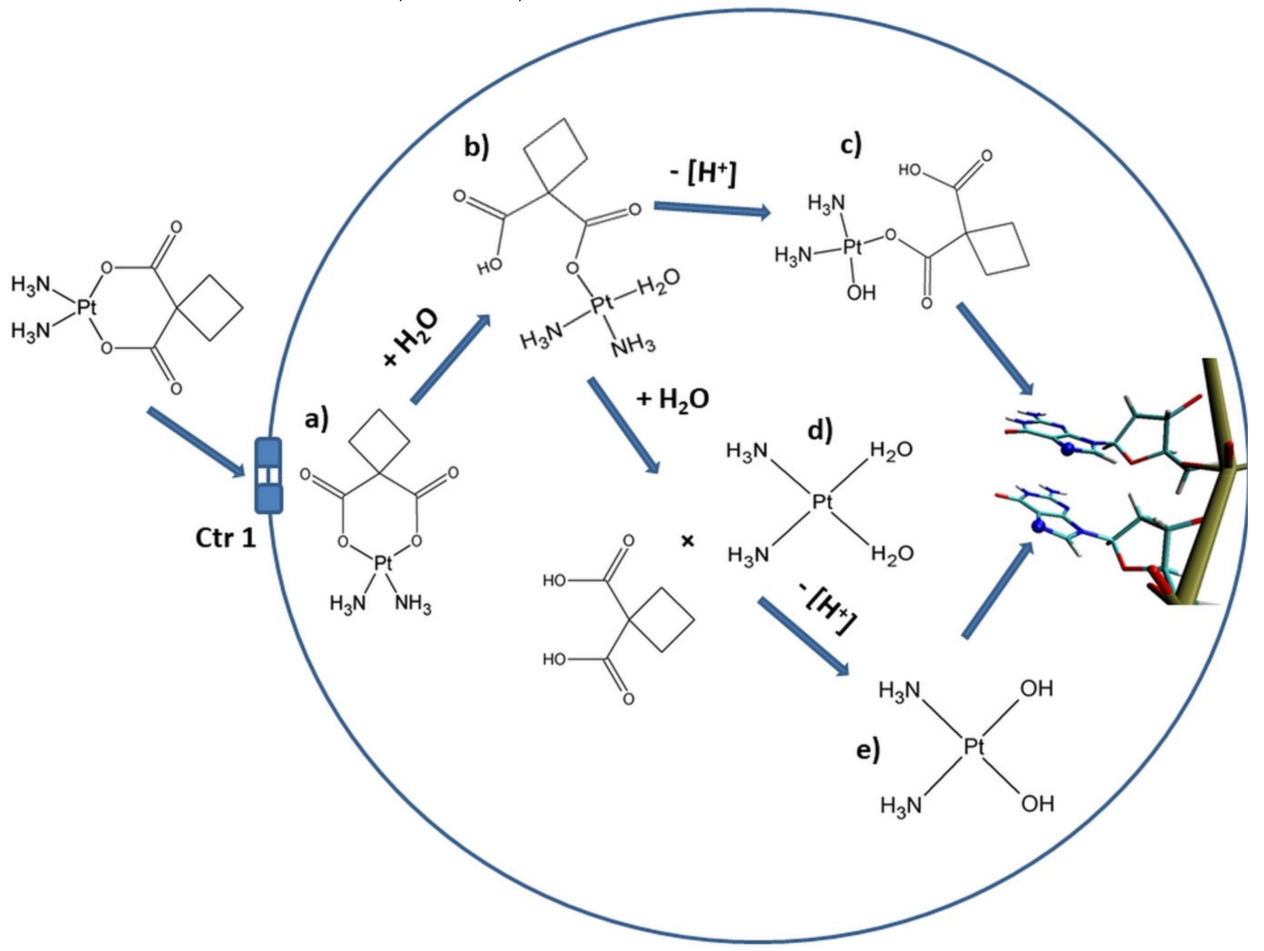
The pathway of carboplatin and anticancer activity. Ctr1 is a high-affinity copper transporter. The following labels correspond to (**a**) carboplatin (**b**) carboplatin after hydrolysis of one bond between Platinum and 1,1-cyclobutanedicarboxylate (**c**) hydroxyl derivative of mono hydrolyzed carboplatin molecule (**d**) di-aqua cis-platin (**e**) di-hydroxyl cis-platin.

Carboplatin as a nature prodrug does not interact with nucleobases of DNA or vitamins in its native form. To be activated, the carboplatin must cross the cell membrane. Inside the cell, the molecule undergoes hydrolysis. CTR (high-affinity copper transporter) as a copper transporter [23,24,25,26] primarily participates in the transport of carboplatin through the cell membrane inside the cell where the molecule undergoes the hydrolysis of 1,1-cyclobutanedicarboxylate (Scheme 1). The decomposition of carboplatin in water is expected to take place through a biphasic mechanism with a ring-opening process followed by the loss of the malonato ligand (Scheme 1b). In this way, it becomes positively charged. The bond breakage between the platinum and the oxygen atoms in the carboplatin leads to the formation of a molecule with a total charge of one (Scheme 1b), next in the presence of water the molecule undergoes further hydrolysis and the [(NH_3_)_2_Pt(H_2_O)_2_]^2+^ molecule (di-aqua cis-platin) is formed (Scheme 1d). Deprotonation of such a molecule leads to the formation of an (NH_3_)_2_Pt(OH)_2_ molecule (di-hydroxyl cis-platin) (Scheme 1e), which undergoes dehydroxylation to ([Pt(NH_3_)_2_(OH)]^+^). Such molecules can generate the formation of adducts of platinum by interacting with nucleophilic molecules within the cell, such as DNA, RNA, and proteins [27]. Through covalent binding of carboplatin to the N_7_ site of purine bases, DNA-protein or DNA-DNA interactions are formed (Scheme 1).

**Figure 2:**
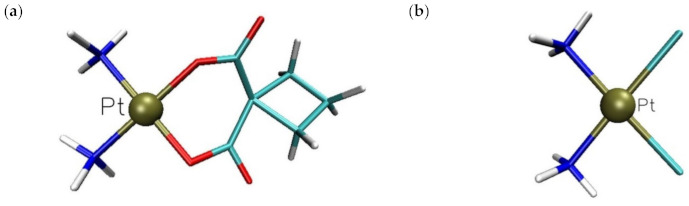
Structure of carboplatin (**a**) and cisplatin (**b**).

Figure 2 represents the structures of carboplatin and cisplatin [28]. When pharmacodynamically comparing carboplatin with cisplatin, it has fewer side effects than its precursor cisplatin. Carboplatin also has lower potency, which may be due to differences in the rate of DNA adduct formation. These toxicity differences are probably related to the low reactivity rate of carboplatin with nucleophiles, as 1,1-cyclobutanedicarboxylate is a poorer leaving group than chloride [9].

**Figure 3:**
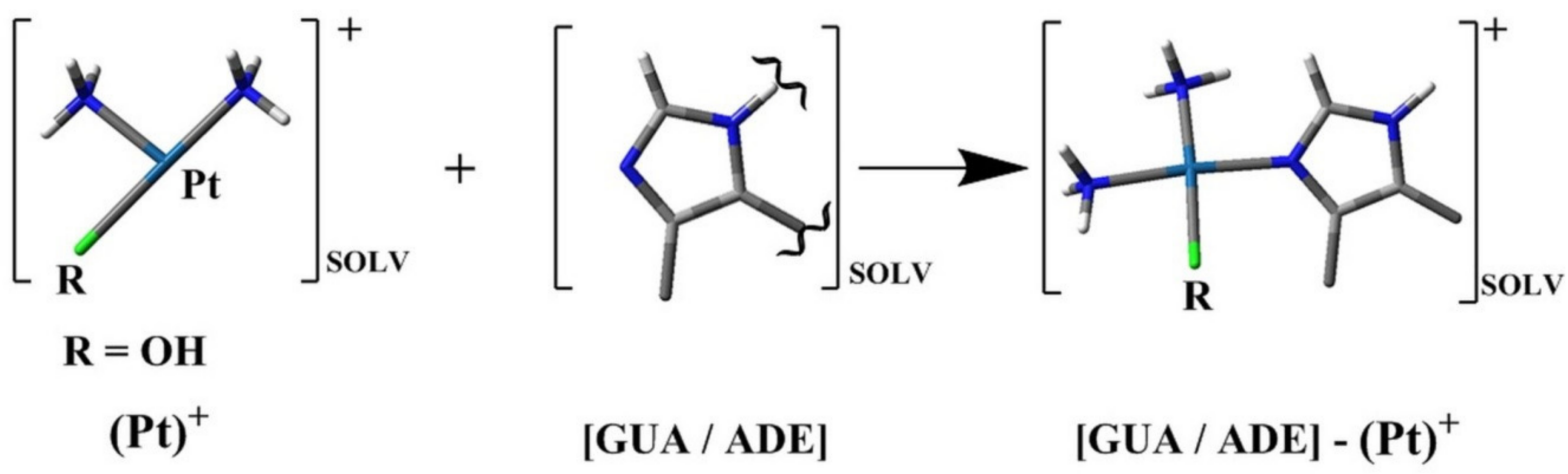
Scheme of the interaction of carboplatin with nucleobases.

The connection between DNA and carboplatin causes changes in DNA (Figure 3), which inhibits the process of DNA replication that generates replication errors, along with the accumulation of cells in the G2/M phase and induction of apoptosis [29]. The formation of connections between carboplatin and DNA strands causes cytotoxicity.

**Figure 4:**
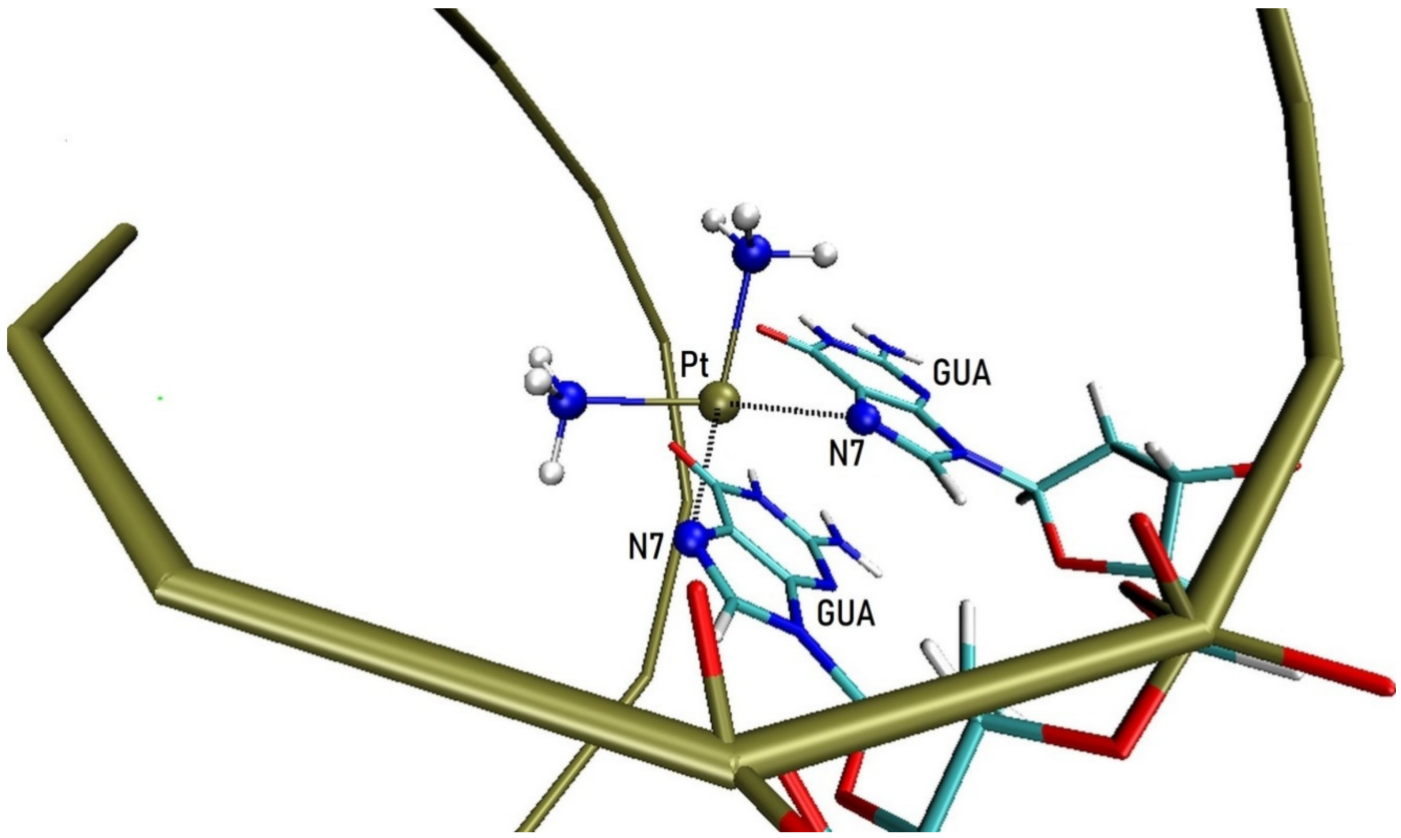
The crosslink of carboplatin with Guanine (GUA) on the DNA.

These processes lead to the creation of one and two positively charged complexes that react with the DNA bases forming a mono-functional adduct. The electron-rich site on DNA is the N_7_ position on Guanine (Adenine) and 1, 2-GpG (1, 2-ApG). The majority of adducts formed by binding of carboplatin to Guanine (or Adenine) are intra-strand crosslinks (Figure 4).

In this way, carboplatin forms Guanine-Guanine, Adenine-Adenine, or Adenine-Guanine adducts what leads to DNA strand distortion [30,31].

### 1.3. The Motivation of Work

It is well known that carboplatin passes through the cell membrane to the inside of the cell where the molecule undergoes the hydrolysis of 1,1-cyclobutanedicarboxylate (Scheme 1) [23,24,26]. In this way, it becomes positively charged. Such highly reactive molecule significantly contributes to the formation of adducts of platinum by interacting with nucleophilic molecules within the cell, such as DNA, RNA, and proteins. As it is also well known that carboplatin, as well as cisplatin [1,4,7,10], have anticancer activity by interacting with DNA and inducing the programmed cell death, the following question is addressed here: how the presence of competitive compounds, such as vitamins containing aromatic rings with lone-pair orbitals analogous to N_7_ in purine, will influence the therapeutic effect of carboplatin?

**Figure 5:**
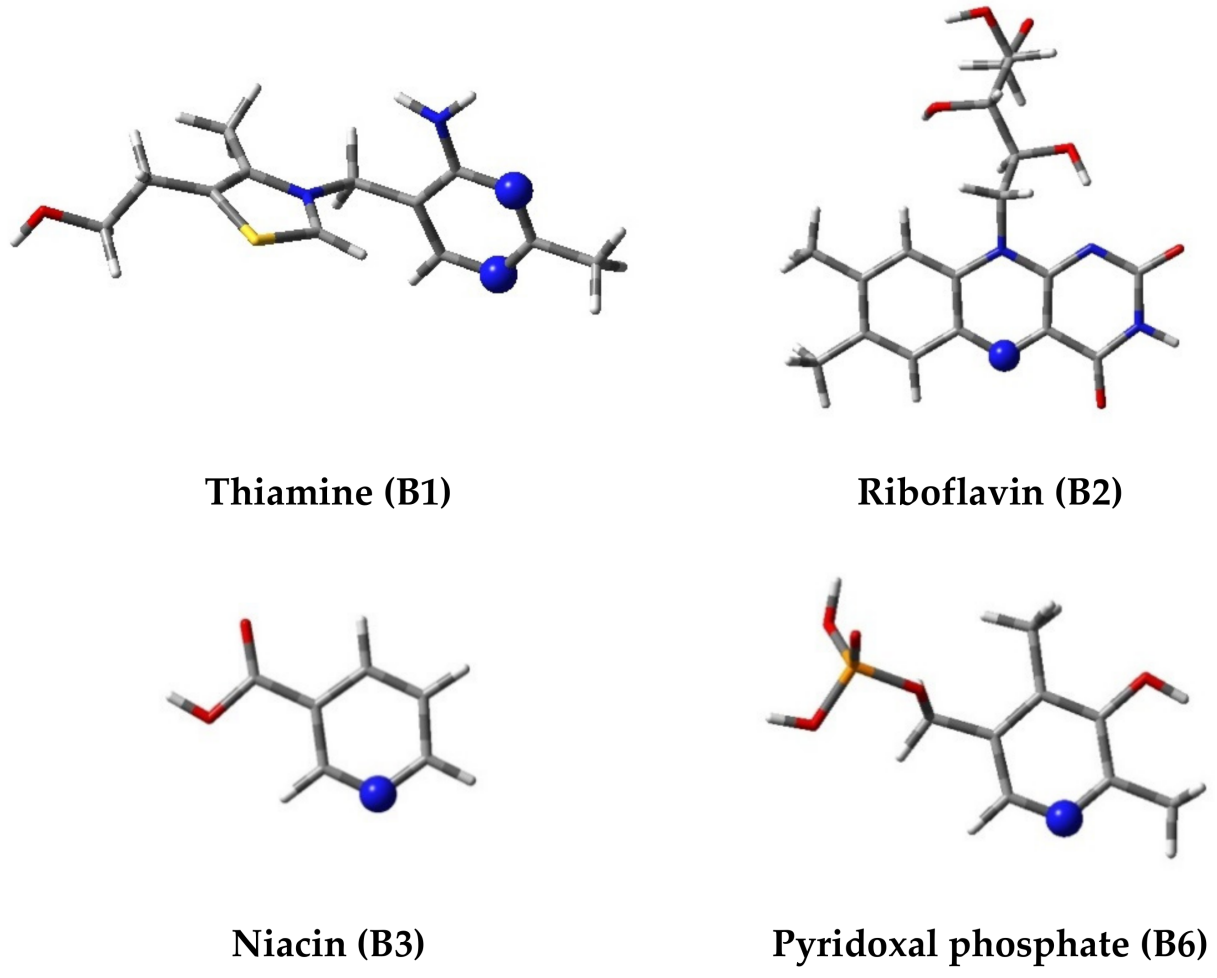
The B vitamins.

The impulse for this work is the observation of the reduction of cisplatin effectiveness when administered to patients suffering from cancer in situations of regular consumption of fresh vegetable juices, particularly carrot and beetroot during treatment, which are rich in Thiamine (B1), Riboflavin (B2), Pyridoxal phosphate (B6) and Niacin (B3) [32] (Figure 5). As this problem was described in an earlier work [33], one should ask another question: how will drinking these juices by the patient affect the cancer treatment during carboplatin therapy?

Based on the literature, it can be said that high levels of some B vitamins can cause cancer or accelerate its development [34,35,36]. However, it is widely accepted that eating meals rich in vitamins and minerals, such as carrot and beetroot juice, reduces the likelihood of cancer, prevents it from metastasis, and may even help with cancer therapy. The concentration of B vitamins at the cellular level depends on the dose concentration of these vitamins and the type of transport [37,38,39]. On the other hand, after cisplatin chemotherapy, the patient receives small daily doses of vitamin B6 (2.5 mg per day), because this compound increases the bioavailability of magnesium ions, which in turn causes a decrease in the toxic effect of cisplatin during anticancer therapy, in particular the reduction of the neuropathy effect [40,41].

Hence, the aim of this work is a theoretical and experimental study on the initial Pt-N_7_(N_1_) bond formation with vitamins from the B group and their comparison with values characterizing native purines.

## 2. Results

### 2.1. In Silico Study

**Figure 6:**
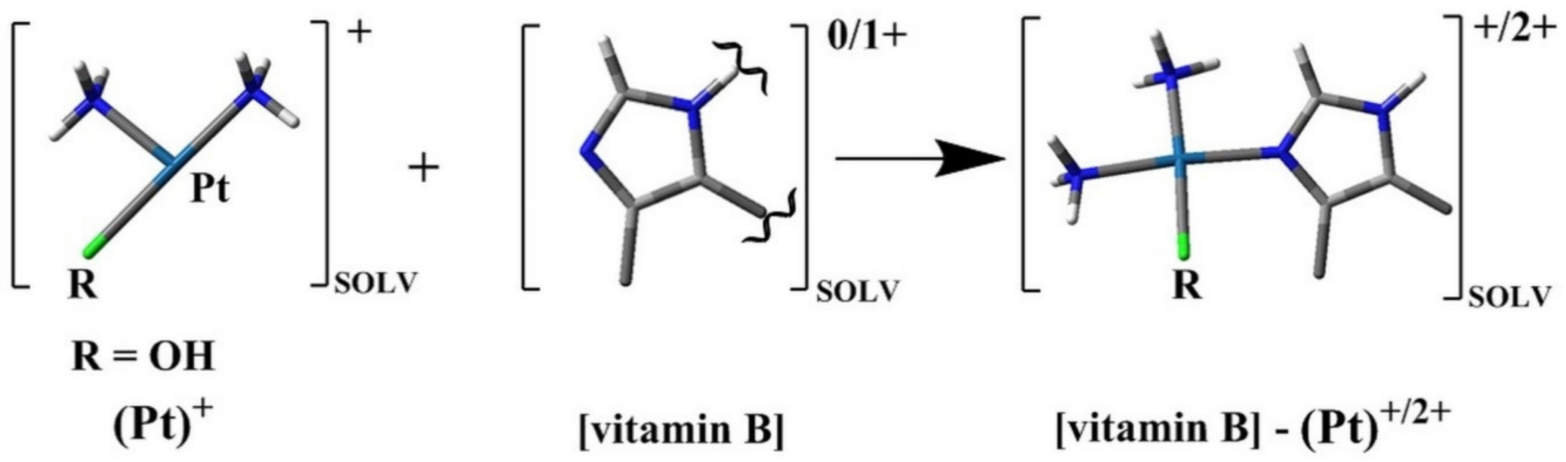
Scheme of complexes of B vitamins with Pt(NH_3_)_2_(OH)^+^ of carboplatin. The only vitamin with a positive charge is Thiamine (vitamin B1).

In our study, there were analyzed the reactivities of ([Pt(NH_3_)_2_(OH)]^+^) derivatives of carboplatin (Figure 3 and Figure 6), with the following compounds belonging to the group of B vitamins, namely Thiamine (vitamin B1), Niacin (vitamin B3), Riboflavin (vitamin B2) and Pyridoxal phosphate (vitamin B6) (Figure 3).

All these B vitamins, as well as Guanine and Adenine, contain aromatic rings with lone-pair orbitals analogous to N_7_ in purine. The similar structure of B vitamins enables them to react in a similar way as Guanine or Adenine, so they are certainly competition for nucleobases. Besides, Thiamine (vitamin B1) not only has a N_7_ atom in purine, but also a second nitrogen atom N_1_. Based on an earlier study [33], it is known that affinities of all analyzed vitamins to cis-Platinum are smaller if compared to Guanine (GUA). However, it has been found that for all chloroaqua and diaqua platinumed complexes the estimated values of Gibbs free energy of reaction ΔG_r_ are negative (Table 1 and Table 2) [33].

Contrary, it has been found that for the two of four ([Pt(NH_3_)_2_(OH)]^+^)-vitamin B complexes the estimated values of Gibbs free energy of reaction ΔG_r_ are positive (Table 3). However, for the complexes of carboplatin with Guanine and Adenine the estimated values of Gibbs free energy of reaction ΔG_r_ are negative, which was, of course, expected [33]. Only for complexes of carboplatin with vitamin B3 (Niacin) and B6 (Pyridoxal phosphate), the energies are negative (Table 3).

Hence, it can be concluded that part of considered vitamins, being a component of beet and carrot juice, cannot freely react with carboplatin, which in consequence cannot possibly lead to a reduction of the therapeutic effectiveness of this drug, which is the opposite of cisplatin. The exceptions are vitamins B3 and B6, which may interact with carboplatin.

### 2.2. Experimental Analysis

Quantum-mechanical calculations show that vitamin B6 (pyridoxine hydrochloride) has the highest affinity for carboplatin (Table 3) with the value of Gibbs free energy of reaction equal to −12.96 kcal/mol. That is why the complexation of carboplatin with this vitamin was carried out experimentally.

**Figure 7:**
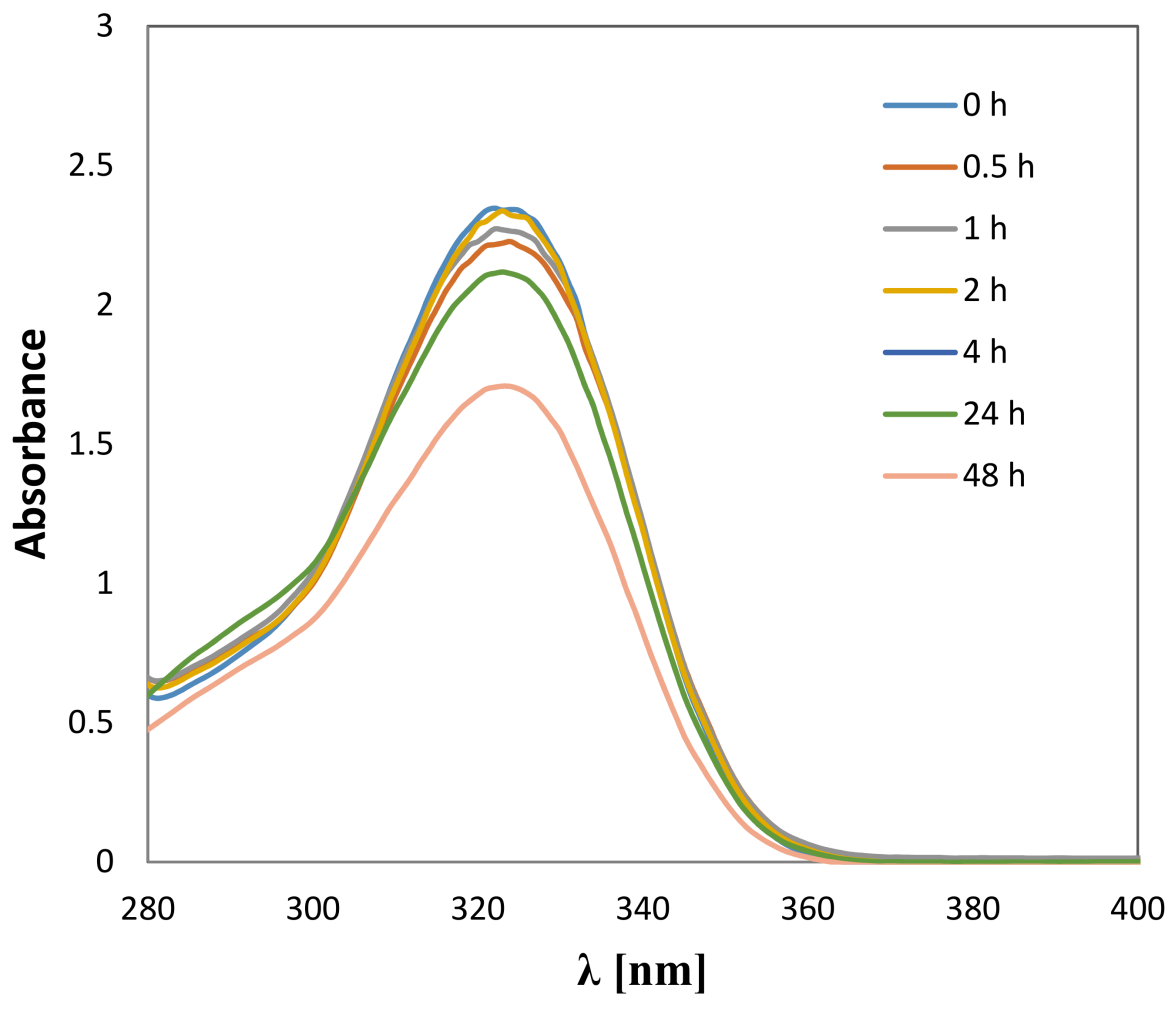
UV-vis absorbance spectrum of vitamin B6 and carboplatin mixture in an incubation buffer (1 mmol L^−1^ phosphate buffer, 4 mmol L^−1^ sodium-chloride, pH 7.4).

Physico-chemical characterization of the interaction of pyridoxine hydrochloride (vitamin B6) and carboplatin was performed by using UV-vis spectroscopic techniques, in the wavelength range from 190 nm to 500 nm (Figure 7).

In an incubation buffer, the maximum absorbance of the vitamin was obtained at 323 nm wavelength, with absorption equal to 2.928 (Figure 7) what reflects a 0.721 mmol/L concentration of vitamin B6. After adding the solution of carboplatin to the solution of vitamin B6 in buffer, the maximum absorbance for the vitamin decreased to a value of 2.3467 (Figure 7, time 0 h) which reflects a decrease in vitamin B6 concentration to a value of 0.624 mmol/l. The decrease of absorbance of pyridoxine hydrochloride is caused by the formation of a vitamin B6-carboplatin complex. After 48 h the concentration of vitamin B6 in the mixture was only 0.515 mmol/L. It is therefore evident that after 48 h, compared to the initial starting point, there is observed a decrease in vitamin B6 concentration by more than 17% after administration of carboplatin solution, and a 28% decrease, relative to the baseline vitamin B6 concentration value.

## 3. Discussion

The affinities of B-vitamins to carboplatin were quantified and compared to interactions with canonical purines (Table 3, Figure 6). For the vitamin with the highest affinity for carboplatin physico-chemical characterization of vitamin B–carboplatin interaction was performed (Figure 7).

Four vitamins from the B group were studied, namely Thiamine (vitamin B1), Niacin (vitamin B3), Riboflavin (vitamin B2), and Pyridoxal phosphate (vitamin B6). All these vitamins have a similar structure as nucleobases, having a nitrogen atom with lone electron pairs in their structure, which one would suppose allows them to form a complex with carboplatin (Figure 5). For all considered compounds, the computed values of Gibbs Free Energies of reaction (ΔG_r_) were compared with values for cisplatin [33] (Table 1 and Table 2). It has been found that for two ([Pt(NH_3_)_2_(OH)]^+^)-vitamin B complexes the estimated values of Gibbs free energy of reaction ΔG_r_ were positive. However, for the complexes of carboplatin with Guanine and Adenine the estimated values of Gibbs free energy of reaction ΔG_r_ were negative, which was of course expected (Table 3). Only for complexes of carboplatin with vitamins B3 (Niacin) and B6 (Pyridoxal phosphate), the affinity indicates a spontaneous reaction (Table 3). It could be concluded that the part of the considered vitamins, being a component of beet and carrot juice, cannot freely react with carboplatin, which in consequence, cannot possibly lead to a reduction of the therapeutic effectiveness of this drug, what is the opposite of cisplatin. The exceptions being vitamins B3 and B6, which may interact with carboplatin.

Because the quantum-mechanical calculations show that vitamin B6 (pyridoxine hydrochloride) has the highest affinity for carboplatin, the complexation of carboplatin with this vitamin was carried out experimentally. After adding the solution of carboplatin to the solution of vitamin B6 in buffer, the maximum absorbance for this vitamin decreased relative to its baseline value.

## 4. Materials and Methods

### 4.1. Gibbs Free Energy Calculation

The geometries of all compounds were optimized at the B3LYP/6–31G** level of theory using Gaussian 09 [42]. The platinum atoms were described with the use of a lanl2dz basis set including relativistic effective core potentials, necessary in the case of heavy atoms [43]. The vibrational frequency calculations and vibrational entropy corrections (room temperature) were estimated at the same level of theory. The calculations including solvation effects were realized with the use of a self-consistent reaction field (SCRF) approach [44] based on accurate numerical solutions of the Poisson–Boltzmann equation [45]. In all computations including the PCM continuum (the Polarizable Continuum Model) model the water dielectric constant 78 and radii Bondi [46] were applied. The chemical affinity was computed by adding contributions of ZPE corrections (Zero-Point Energy), thermal corrections to the enthalpy and entropy terms, as well as continuum solvation free energies.

### 4.2. Spectroscopic Measurements 

**Figure 8:**
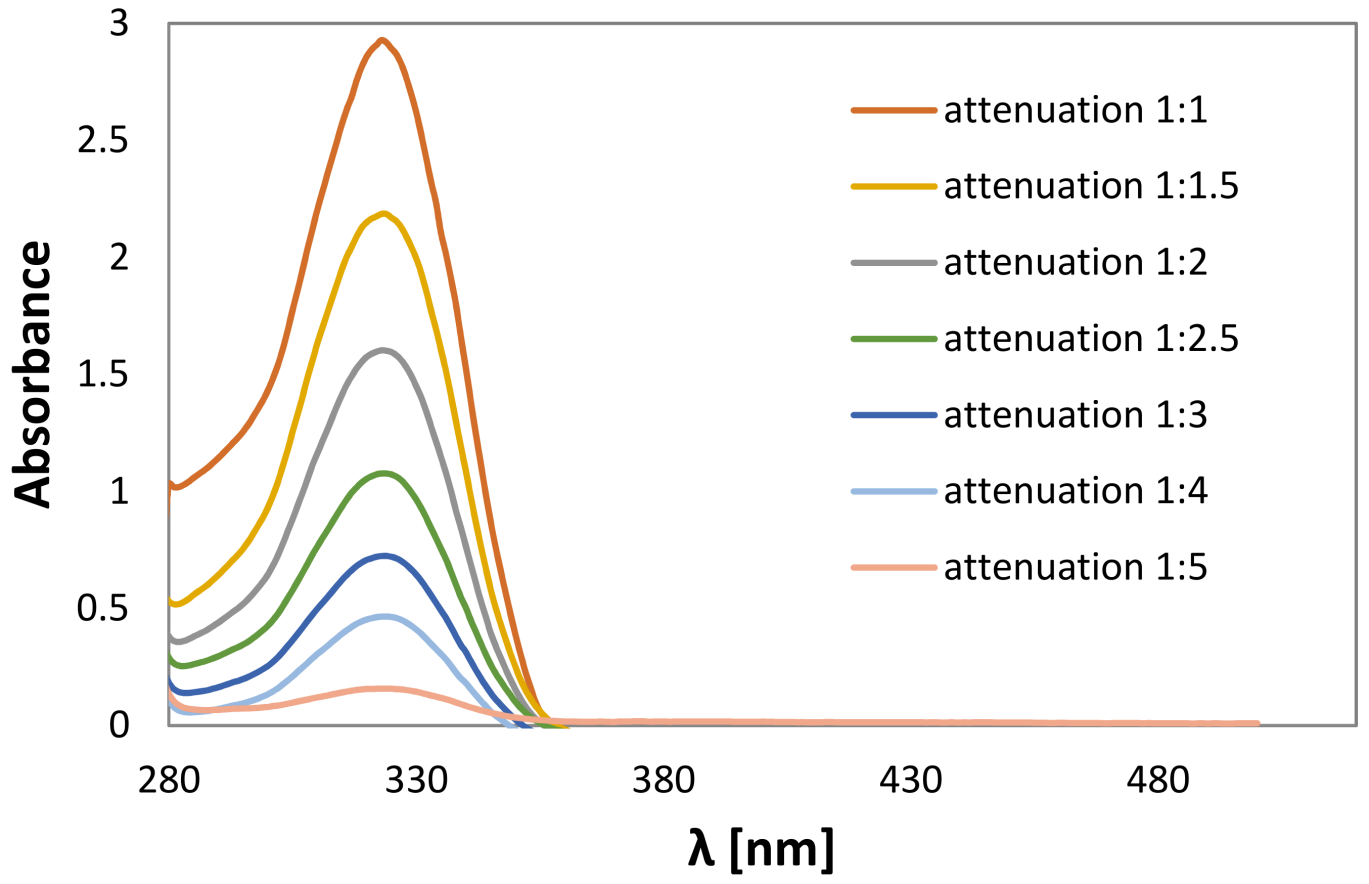
UV-vis absorbance spectrum of vitamin B6 (Pyridoxal phosphate).

**Figure 9:**
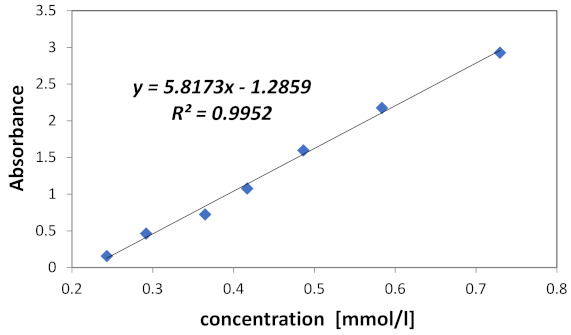
A standard curve for vitamin B6 (Pyridoxal phosphate).

In the experimental part of the study, the following reagents were used: pyridoxine hydrochloride was purchased from Pol-Aura, Poland, sodium chloride (pure p.a.), from Avantor, Poland, and carboplatin solution for injection 10 mg/mL from Pfizer Service Company. The phosphate buffer for release was obtained from Chempur, Poland. The concentration of vitamin B6 was measured spectrophotometrically on a Biosens UV-6000 spectrophotometer with a resolution of 1 nm, the absorbance was measured at 323 nm. Both carboplatin (7.3 × 10^−4^ mol × L^−1^) and vitamin B6 (14.6 × 10^−3^ mol × L^−1^) were prepared in the incubation buffer (1 mmol × L^−1^ phosphate buffer, 4 mmol × L^−1^ sodium-chloride, pH 7.4). For the solutions of the considered vitamin, a standard curve was established based on the set of dilutions of the stock solution (Figure 8 and Figure 9). During the measurements, the vitamin B6 (14.6 × 10^−3^ mol L^−1^) was incubated at 37 °C with carboplatin in a ratio of 2:1. Aliquots of this mixture were taken after 0 h, 0.5 h, 1 h, 2 h, 4 h, 24 h, and 48 h incubation.

## 5. Conclusions

The affinity of B-vitamins to carboplatin was studied both using computational chemistry, as well as UV-vis spectroscopy. The obtained results were compared with the reference values of nucleobases of DNA. The affinity of the complexes of carboplatin with vitamins B3 (Niacin) and B6 (Pyridoxal phosphate) indicates a spontaneous reaction. In spectroscopic studies, it was revealed that after complexation of B6 vitamin and carboplatin, the decrease of maximum absorbance for the vitamin relative to the baseline is observed.

## Figures and Tables

**Table 1:** The energetic characteristics of the cis-Pt-Chloroaqua reactions with derivatives of vitamin B. All energies are given in kcal/mol. Symbol Pt* stands for Pt(NH_3_)_2_Cl [33].

Number of Reaction	Reaction	Δ*G_r_*
1	cis[Pt*]^+^ + B1(N_7_) → cis[Pt*~B1(N_7_)]^+^	−18.90
2	cis[Pt*]^+^ + B1(N_1_) → cis[Pt*~B1(N_1_)]^+^	−20.89
3	cis[Pt*]^+^ + B2(N_7_) → cis[Pt*~B2(N_7_)]^+^	−3.75
4	cis[Pt*]^+^ + B3(N_7_) → cis[Pt*~B3(N_7_)]^+^	−24.90
5	cis[Pt*]^+^ + B6(N_7_) → cis[Pt*~B6(N_7_)]^+-^	−15.61
6	cis[Pt*]^+^ + GUA(N_7_) → cis[Pt*~GUA1(N_7_)]^+^	−28.17

**Table 2:** The energetic characteristics of the cisPt-diaqua reactions with derivatives of vitamin B. All energies are given in kcal/mol. Symbol Pt** stands for Pt(NH_3_)_2_(H_2_O) [33].

Number of Reaction	Reaction	Δ*G_r_*
1	cis[Pt**]^2+^ + B1(N_7_) → cis[Pt**~B1(N_7_)]^2+^	−20.23
2	cis[Pt**]^2+^ + B1(N_1_) → cis[Pt**~B1(N_1_)]^2+^	−22.64
3	cis[Pt**]^2+^ + B2(N_7_) → cis[Pt**~B2(N_7_)]^2+^	−7.84
4	cis[Pt**]^2+^ + B3(N_7_) → cis[Pt**~B3(N_7_)]^2+^	−27.28
5	cis[Pt**]^2+^ + B6(N_7_) → cis[Pt**~B6(N_7_)]^2+^	−24.66
6	cis[Pt**]^2+^ + GUA(N_7_) → cis~[Pt**~GUA(N_7_)]^2+^	−37.58

**Table 3:** The energetic characteristics of the ([Pt(NH_3_)_2_(OH)]^+^) reactions with derivatives of vitamin B. All energies are given in kcal/mol. Symbol Pt*** stands for Pt(NH_3_)_2_(OH).

Number of Reaction	Reaction	Δ*G_r_*
1	carbo[Pt***]^+^ + B1(N_7_) → carbo[Pt***~B1(N_7_)]^+^	149.53
2	carbo[Pt***]^+^ + B1(N_1_) → carbo[Pt***~B1(N_1_)]^+^	144.60
3	carbo[Pt***]^+^ + B2(N_7_) → carbo[Pt***~B2(N_7_)]^+^	233.04
4	carbo[Pt***]^+^ + B3(N_7_) → carbo[Pt***~B3(N_7_)]^+^	−6.3
5	carbo[Pt***]^+^ + B6(N_7_) → carbo[Pt***~B6(N_7_)]^+-^	−12.96
6	carbo[Pt***]^+^ + ADE(N_7_) → carbo[Pt***~ADE(N_7_)]^+^	−26.46
7	carbo[Pt***]^+^ + GUA(N_7_) → carbo[Pt***~GUA(N_7_)]^+^	−30.89

## Data Availability

All data is in the article.
